# Moving forward better marketing for a better world: a path for new research opportunities

**DOI:** 10.1007/s43039-023-00072-5

**Published:** 2023-04-14

**Authors:** Yanina Rashkova, Ludovica Moi

**Affiliations:** Department of Economics and Business, University of Cagliari, Viale Sant’Ignazio 74, 09123 Cagliari, Italy

**Keywords:** Better marketing for a better world, Research agenda, Literature review

## Abstract

The topic of marketing as a source of a “better world” is gaining rising importance in academia and practice. It represents an opportunity to move towards the development of a more prosperous, fair, and equitable society. Nevertheless, questions remain about how organizations could comprehensively form and nurture marketing for good. In this paper, we display a critical review of the most widely accepted theoretical approaches related to better marketing for a better world (BMBW) to seek new research perspectives. We contribute to extending prior literature by presenting its main criticalities, articulating them around three clusters of shortcomings in view of how recent literature is evolving. Based on this analysis, we then recommend avenues for future research and associated research questions to stimulate and advance further scholarly investigations.

## Introduction

Scholars and practitioners often ask: “Can marketing help the world to be better?” Ideally, the answer would be *“Absolutely, yes!”* Being at the front of all market exchanges, guided by parties looking to solve needs efficiently and effectively, and empowered by recent technological progress, marketing has the power to improve lives and benefit the world at large (Chandy et al., 2021; Marino et al., 2020; Sheth et al., 2011).

The idea of marketing as a way for a better world is not new (Drucker, 1969). Several marketing scholars endorse the idea that the primary purpose of marketing is to drive positive changes in the world and society (Chandy et al., 2021). This perspective supports that marketing has the potential to shift routinized dynamics both within and outside the organization to strive toward achieving a better place to work and live in. Frequently used as an umbrella term, better marketing for a better world (BMBW) encompasses the belief that marketing activities may have a positive impact beyond organizational frontiers, and, in the long run, they enhance the welfare of the world’s stakeholders and institutions, such as businesses, customers, and the society at large (Chandy et al., 2021). In this conception, for instance, by emphasizing the long-lasting nature of high-end products, consumers are more likely to overcome product durability neglect and buy fewer but better high-end products (Sun et al., 2021). Similarly, prior research found that to overcome consumer resistance to sustainability interventions, companies and governments should not target individual behaviors but social practice changes (Gonzalez-Arcos et al., 2021).

Importantly, the concept of BMBW differs from similar marketing approaches, such as social marketing, which applies marketing techniques to social problems and mostly practiced in public entities without lucrative scope (Kotler & Zaltman, 1971). Similarly, a green marketing approach may be related to BMBW whenever it creates both environmental, firm, and customer values (Dangelico & Vocalelli, 2017; Gopaldas, 2015).

To date, there is cross-fertilizing research on marketing concepts across literature endorsing BMBW philosophy, from the early conceptualization of societal marketing (Kotler, 1999), to the more recent ones like the concept of positive marketing (Gopaldas, 2015; Lerman & Shefrin, 2015; Stoeckl & Luedicke, 2015), mindful marketing (Hagenbuch & Mgrdichian, 2020; Malhotra et al., 2012; Sheth et al., 2011), and socially responsible marketing (Laczniak & Shultz, 2020), among others. Although the plethora of empirical and theoretical works on this topic, questions remain about how to comprehensively form and nurture BMBW in organizations. As marketing research has greatly advanced in the last few years, its theoretical ground has become more complex, bringing a range of overlaps and similarities between the different BMBW marketing approaches (Bayraktar, 2020; Tadajewski 2016). Moreover, despite previous studies that have contributed to assisting businesses in practicing better world marketing, little is known about marketing’s role in improving—or harming—our world (Chandy et al., 2021). As such, it still lacks unanimous theoretical consent on how organizations should apply BMBW (Marino et al., 2020; Wilkie & Moore, 2012).

To fill these gaps, the main objective of this paper is to identify missing research areas in the literature and to craft a future research agenda to advance BWBM research and practice. Performing a conceptual literature review (Webster & Watson, 2002), this study aims to answer the following research question: “How to advance research on better marketing for a better world?”

To answer our research question, we first provide a review of the key features surrounding the main concepts associated with BMBW to highlight the progress scholars have made regarding this topic. Based on this analysis, we then identify three main shortcomings found in prior literature. Drawing on the identified shortcomings, we portray some avenues for future research to enhance marketing theory and strengthen our understanding of how marketing can be implemented to achieve greater sustainability for business and society.

Today’s world is still far from an ideal and prosperous place to live. The global epidemiological situation provoked by Covid-19 and the humanitarian crisis due to the war in Ukraine have further exacerbated protracted economic and environmental crises (White et al., 2022). Therefore, it is more than evident the need for greater attention to BMBW. Moreover, promoting BMBW marketing research is paramount to business; as Drucker (1969) pointed out, a healthy business and a diseased society are incompatible. Finally, marketers should ensure that the theoretical understanding of marketing for good is clear to guarantee the successful development of future marketing research and practice led by young scholars.

## Literature review on better marketing for a better world

To analyze the main literature on BMBW and identify the key theoretical underpinnings of the different marketing approaches proposed by scholars on this topic, we followed the methodology of Webster and Watson (2002) and developed a concept-driven matrix. In constructing our literature review, we specifically focused on marketing approaches that fully meet the definition of BMBW, i.e., “marketing activities and ideas that impact outcomes beyond just what is good for the financial performance of firms: BMBW emphasizes marketing’s role in enhancing the welfare of the world’s other stakeholders and institutions” (Chandy et al., 2021, p.1).

When choosing BMBW approaches, we proceeded as follows. First, we took a close look at the leading marketing journals (e.g., Journal of Marketing, Journal of the Academy of Marketing Science), reviewing the literature on sustainable marketing practices in a broad sense from 1970 forward. We further performed a keyword search in the electronic databases (e.g., Web of Science, ELSEVIER, and EBSCO) using “sustainability” and/or “societal” and/or “responsible” (and variations on the word spelling) as keywords, followed by an Internet search on Google Scholar. The guiding criterion for inclusion was the focus of the marketing concept on profit, people, and the planet at the same time, or similarly marketing concept profiting from sustainable, social, and environmental practices. For example, we excluded a concept whenever it only targeted the social agenda. Once we completed the initial search, we shared the identified concepts with senior marketing scholars to ensure that we covered all existing related concepts.

In our review, we, therefore, included the following concepts associated with BMBW: societal marketing, corporate societal marketing, mindful marketing, positive marketing, and socially responsible marketing. To better understand the BMBW, we classified these concepts by considering the following categories: axiological basis (the presence of discourse concerned with the values by which the concept upholds), supported theory (the presence of theoretical basis), practice relatedness (the strength of practical implementations), and the main instruments (approaches used to reach BMBW). Table 1 provides the synthesis of the above.

**Table 1:** Concept matrix of Better Marketing for Better World concepts

Main authors	Marketing Concept	Theory-grounded		Axiological basis		Practice related			Main BMBW instruments				
		Yes	No	Yes	No	Yes	No	Partially	All marketing activities	Product development	Marketing Innovation	Consumer consumption	Corporate citizenship, stakeholder orientation, and social/ecological sustainability
Abratt & Sacks 1988	Societal marketing		X		X			X		X			
Bayraktar et al., 2020	Mindful entrepreneurial marketing	X			X		X		X				
Crane & Desmond 2002	Societal marketing		X	X			X			X			
Drumwright & Murphy 2001	Corporate societal marketing		X		X			X	X				
Dhaka et al., 2021	Corporate societal marketing		X		X	X			X				
El-Ansary 1974	Societal marketing		X		X		X			X			
Gopaldas 2015	Positive marketing		X		X	X				X	X		
Hagenbuch & Mgrdichian 2020	Mindful marketing	X			X		X					X	
Hoeffler & Keller 2002	Corporate societal marketing		X		X	X			X				
Kotler, 1971	Societal marketing		X		X			X		X			
Krush et al., 2015	Positive marketing		X		X	X				X	X		
Laczniak & Murphy 2019	Socially responsible marketing	X		X				X		X		X	X
Laczniak & Shultz 2020	Socially responsible marketing	X		X				X		X		X	X
Lerman & Mejia 2018	Positive marketing		X		X		X			X	X		
Lerman & Shefrin 2015	Positive marketing		X		X		X			X	X		
Sheth et al., 2011	Mindful marketing	X			X			X				X	
Sheth & Sisodia 2006	Mindful marketing	X			X			X				X	
Schwartz 1971	Societal marketing		X		X		X			X			
Uslay & Erdogan 2014	Mindful entrepreneurial marketing	X			X		X		X				
Ward & Lewandowska 2006	Societal marketing	X			X		X			X			

Source: own elaboration

### Societal marketing

The emergence of BMBW is frequently associated with the development of the concept of societal marketing, the authors of which claim that companies need to focus on society and not on individual needs and desires (El-Ansary, 1974; Schwartz, 1971; Ward & Lewandowska, 2006). As argued by the guru of marketing Kotler (1971), to sustain growing consumerism businesses should reexamine their social roles by making long-run social welfare a priority. By developing the concept of societal marketing, he holds that “the organization’s task is to determine the needs, wants, and interests of target markets and to deliver the desired satisfactions more effectively and efficiently than competitors in a way that preserves or enhances the consumer’s and the society’s well-being” (Kotler, 1999, p. 4).

While arguing at that time whether marketers have the entitlement to determine what is right for society (Schwartz, 1971), the authors fully agree that economic goals should not prevail over social programs (Schwartz, 1971; Ward & Lewandowska, 2006). They also support the belief that what is good for society, in the long run, may also be good for business. In practice, societal marketing avoids messages that leverage buyers’ emotions or exploit unsophisticated elements of the population (Schwartz, 1971).

In this vein, organizations mostly reached BMBW through developing desired products that seek to address contemporaneously immediate consumer satisfaction and long-term consumer welfare (Kotler, 1971). According to prior literature, societal marketing particularly emphasizes the prominent role of product development (Schwartz, 1971). By defining the type of consumer benefits, all products can be divided into four groups: deficient products that provide neither short nor long-term benefits; salutary products that offer low satisfaction but high long-term social benefits; pleasing products that offer immediate satisfaction but have low societal benefits; and desirable products that contemporaneously address consumers’ desire and social welfare (Kotler, 1971). Empirical evidence of a successful application of societal marketing can be observed in the case of The Body Shop, a British cosmetic, skincare, and perfume company that champions human and civil rights as well as animal and environmental issues (The Body Shop, 2022). By using ingredients that do not harm people and the environment, The Body Shop satisfies the customers’ needs while also ensuring long-term social benefits.

### Corporate societal marketing

Over time, societal marketing has grown into the concept of corporate societal marketing (CSM), which takes into consideration all marketing activities, including communication and strategy, to bring about societal benefits (Drumwright & Murphy, 2001). CSM suggests that it is not enough for a company to think only about the monetary exchange with customers, but the marketing strategies should also concentrate their efforts on delivering value to the customers in a manner that improves the well-being of the customer as well as the society at large (Dhaka et al., 2021). In fact, corporate societal marketing actions should use any organizational resources and target at least one non-economic result, such as supporting the local community or raising awareness of social issues (Hoeffler & Keller, 2002).

Prior literature highlights that CSM can take multiple forms varying on the type of resources employed, employee involvement, and budget sources (Drumwright & Murphy, 2001). Well-known examples of CSM are charitable campaigns implemented in collaboration with an existing NGO that aim at donating profits derived from the purchase of a company’s products to a social cause (Drumwright & Murphy, 2001). Also, companies may decide to create a new cause program and then associate it with either the corporate name or one of their product brand’s names (Hoeffler & Keller, 2002). A practical example of corporate societal marketing may be the case of Ariel, a detergent brand owned by Procter and Gamble. Notably, Ariel runs special fund-raising campaigns for less privileged classes of the world, particularly in developing countries. It also contributes to sharing its profits from every bag sold for societal development (Procter & Gamble, 2022). By doing so, Ariel builds strong brand equity (Hoeffler & Keller, 2002) while also advancing human development by contributing resources and organizational know-how useful for overcoming societal issues.

In this vein, BMBW can be achieved by simultaneously creating value for society and the company. Particularly, from a company’s point of view, supporting a socially significant cause leads to increased brand loyalty (Hoeffler & Keller, 2002), which guarantees long-term sustainability. From a social perspective, marketing efforts based on organizational know-how and collective knowledge support social efforts to overcome social problems (Chattananon et al., 2007).

### Positive marketing

Following the conceptualization of CSM, it emerged the concept of positive marketing. According to scholars, positive marketing refers to any marketing activity aimed at creating value for the firm, its customers, and society so that the involved parties are better off than before the market exchange (Gopaldas, 2015; Lerman & Shefrin, 2015). Supporting the idea of societal marketing, the contributing authors fully acknowledge that companies never give up profits, but they may redirect their focus to win-win situations. Thus, they make a profit by satisfying various aspects of customers’ needs through positive marketing means (Lerman & Mejia, 2018; Lerman & Shefrin, 2015).

Organizations can reach BMBW through marketing innovation that may take the form of material, meaning, or practice innovations (Gopaldas, 2015). An example of positive marketing empowered by material and practice innovation is the famous case of Patagonia, high-end outdoor apparel that continuously greens its supply chain and advocates for anti-consumerism, calling for conscious leadership (social value) and offering a lifetime warranty and a free repair service (customer value). By benefiting society and consumers, the company represents the world’s biggest and best-known name in outdoor wear, with annual sales of about $1 billion per year (company value) (Ryan, 2021).

Although some critics claim that positive marketing is an understudied concept of societal marketing (Tadajewski, 2016), it seems that positive marketing extends the understanding of betterment marketing embodied in societal marketing by considering brands as creative, forward-thinking agents in society and highlighting the importance of marketers’ proactive engagement, activist executives, and networked customers (Gopaldas, 2015; Lerman & Shefrin, 2015). Moreover, a positive marketing approach embodies a useful lens to study a successful case of business model innovation in the context of the sharing economy enterprise (Krush et al., 2015).

### Mindful marketing

With the rise of interest in the topics of awareness and individual mindfulness, mindful marketing has been gaining increasing importance both in academia and practice. Perceived as a set of practices that aim to transform consumption into a business and societal opportunity by generating win-win solutions that consider the triple bottom lines of planet, people, and profit (Sheth et al., 2011), mindful marketing relies on the theory of organizational mindfulness, conceptualized around several practices (Weick & Sutcliffe, 2006). Mindful marketing is effective and ethical, creates stakeholder value, and upholds societal values (Hagenbuch & Mgrdichian, 2020). In this perspective, mindful marketing considers the interest of both sellers and buyers without jeopardizing the state of the third, non-included parties (Malhotra et al., 2012). Similarly, with a positive marketing approach, the primary goal of mindful marketers is to seek ways to create win-win strategies that align marketing activities with customers’ needs and avoid being involved in resource waste and unethical practices (Malhotra et al., 2012; Sheth & Sisodia, 2006).

Differently from other approaches, BMBW is reached through mindful consumption, that is, an experience of consumption that is conscious of the consequences of chosen purchases. Notably, “mindful consumption connotes temperance in acquisitive, repetitive and aspirational consumption at the behavior level, ensuing from and reinforced by a mindset that reflects a sense of caring toward self, community, and nature” (Sheth et al., 2011, p. 30). In this perspective, marketers, by leveraging marketing tools such as product, price, promotion, and place, can foster mindful consumption. A practical example of mindful marketing can be marketing techniques aiming to reduce personal consumption. For instance, product-sharing under “product-service systems,” such as car sharing, communal washing centers, and tool-sharing arrangements, is one of the most applied practical examples (e.g., Mont 2004).

Mindful marketing extends to mindful entrepreneurial marketing (Bayraktar et al., 2020; Uslay & Erdogan, 2014). Defined as a set of processes that embed awareness and attention to social, environmental, and economic realities while simultaneously aligning production and consumption to meet a desired financial performance (Uslay & Erdogan, 2014), mindful entrepreneurial marketing involves proactive alertness to identify problems, consciousness for resource leveraging, and value co-creation through creativity and risk management (Uslay & Erdogan, 2014).

### Socially responsible marketing

Taking the macro and normative perspective, the proponents of socially responsible marketing (SRM) similarly rely on the idea that SRM consists of practices and perspectives that are “mandated by an implicit social contract, which requires marketing policies, actions and outcomes to adhere to a corporate [“good”] citizenship that is proactive and non-discretionary” (Laczniak & Shultz, 2020, p. 4). Stakeholder orientation, which recognizes an authentic evaluation of stakeholder claims especially by customer and vulnerable stakeholders, is the foundation of this approach. Further, SRM seeks social and environmental sustainability in all its actions.

According to this perspective, the continuous implementation of corporate citizenship, stakeholder orientation, and social/ecological sustainability enable to reach BWBM. As for the other approaches analyzed in the previous paragraphs, socially responsible marketing has one more objective besides serving products for profit. Notably, marketers have social responsibilities in front of market stakeholders, such as avoiding negative market externalities that may arise from the creation, delivery, and capture of offered products. Moreover, besides common legal obligations, SRM explicitly endorses ethical values (Laczniak & Murphy, 2019), such as avoiding predatory pricing, disclosing conflicts of interest, embracing environmental stewardship, acting transparently, and protecting customer data. It similarly swears to never knowingly harm others via marketing activity (Laczniak & Shultz, 2020). A practical example of socially responsible marketing can be a marketing practice that avoids price discrimination and treats different buyers equally.

Furthermore, it is essential to notice that SMR focuses on thinking holistically about the environmental impacts of marketing activities, as concerns social, ecological, and financial sustainability (Laczniak & Murphy, 2012). In this perspective, SRM is like mindful marketing that highlights the importance of moderate, temperance consumption (Hagenbuch & Mgrdichian, 2020; Malhotra et al., 2012; Sheth et al., 2011). It notably relies on the ethical perception of producing and promoting products (Laczniak & Murphy, 2019) by asking, “Should it be produced?” instead of “How to promote it?”.

## Shortcomings in the existing research on BMBW

Having provided a brief overview of the main concepts associated with BMBW, in this section, we aim to summarize a few critical areas found in prior literature that requires further scholarly attention. Notably, by drawing on the literature discussed above, we primarily highlight the presence of some conceptual similarities between all BMBW approaches and the approved definition of marketing itself. Moreover, despite some advances in the literature on BMBW, we highlight the lack of practical implementation of BMBW. In further sections, we discuss these points in more detail.

### Conceptual similarities and lack of axiological and theoretical basis

As highlighted in previous sections, BMBW approaches frequently overlap in definitions, methodologies, and applied practices. Previous research illustrated, for instance, the intersection of one approach with another, such as the case of positive marketing being a reinvention of societal marketing (Tadajewski, 2016) or mindful marketing being the antecedent of positive marketing (Bayraktar, 2020). Similarly, despite the presence of strong theoretical basis and clear normative stance, “ the concept of SRM is hardly distinguishable from previously proposed approaches and Corporate Social Responsibility in general.

Moreover, as the literature overview has shown, all the proposed marketing concepts seem to agree that marketing should not harm stakeholders and society, but a lack of theoretical grounding makes it difficult to perceive their conceptual differences. Concepts such as positive marketing and societal marketing specifically lack a grounded theoretical basis that delineates the appropriate tools and practices that organizations should apply.

In addition, prior research has shown that all the aforementioned approaches overlook the discussion of the definition of criteria for good, which makes it difficult to understand what axiological basis this or that approach stands on. We argue that the refinement of concepts might bring theoretical clarity, which will prompt the interest of marketing research society and further practical implementation of BWBM.

### Unrelated marketing definition

We also observed one more overlap in the current marketing literature. Notably, the definition of marketing itself, as “communicating (…), creating value for customers, clients, partners, and society at large” (American Marketing Association, 2017), partially encompasses the BMBW idea. Nevertheless, despite the inclusion of better world inclination in the definition of marketing, the society still perceives marketing as a source of artificial stimulation of the desire to buy a product/service that does not necessarily increase societal well-being (Kitchen & Sheth, 2016). Indeed, in the last sixty years, marketing has been often criticized (Kotler, 2017; Stoeckl & Luedicke 2015), such as for deceptive product and pricing practices, an excessive promotion that displaces the human agency and genuine interest in the product and acceleration of harmful consumerism that advances wasteful materialistic lifestyles at the expense of meaningful alternatives, to name just a few. As such, it is unclear at what point the definition of marketing endorses the idea of BMBW. This problem further leads to a dissonance of the marketing definition and its practical perception in society, which hinders the application of marketing practices for good.

### Underexplored practical implementation of BMBW

Although the shift toward more sustainable and inclusive marketing is noticeable in academia, practitioners are late to embrace this shift, as marketing research very frequently lacks the practical implementation of the proposed approaches and methods. As our overview has shown, there is little understanding of how organizations can practice the above concepts.

For instance, despite the impact of societal marketing on various disciplines known today, such as, for example, corporate social responsibility and business ethics in general (Crane & Desmond, 2002), the concept is very narrow, focusing strictly on product development (Abratt & Sacks, 1988). It is unclear what constitutes the application of societal marketing in practice and whether and how it moves beyond product development (Fiore et al., 2016). Similarly, despite the prominence of the mindful marketing concept, there are still few empirical studies evaluating the implementation of mindful marketing techniques.

As such, we miss the discussion within BMBW literature on how organizations can develop marketing practices and capabilities for “good” (Chandy et al., 2021; Marino et al., 2020). It is unclear who the main actors of change implementations are and what resources organizations need to ensure a sustainable movement toward BW practices.

## Moving toward better marketing for a better world: avenues for future research

The importance of BMBW is increasingly evident in current research and practice. In this paper, we have provided an initial analysis of how research has evolved, searching for the development of theories and concepts that account for marketing for good.

However, there are still important research areas that need further theoretical and empirical attention. Working from the brief rationale above, in this last section, we recommend avenues for future research and associated research questions to stimulate and advance further scholarly investigations.

### Avenue 1: overcoming conceptual similarities

As noticed above, the different BMBW approaches overlap. Future research should clarify how and in what these marketing approaches differ or coincide. Moreover, despite the recent interest in marketing for achieving a better world, we still have little knowledge about marketing’s role in improving our world (Chandy et al., 2021; Wilkie & Moore, 2012). This raises research questions such as the following:

- What are the unifying characteristics of the better world marketing approaches? How do they differ?
- How and when can the combination of different better world approaches strengthen the sustainability of marketing practices?
- What axiological perspective guides better world marketing approaches?

All BMBW approaches seem to agree on marketing as a source of a better world. Nevertheless, the way how it may be reached varies. For instance, a positive marketing approach emphasizes the prominence of innovation (Gopaldas, 2015), whereas mindful marketing particularly highlights mindful consumption to reach a sustainable society (Sheth et al., 2011). We suggest applying a method of concept comparison or co-word analysis to delineate the differences and similarities of BMBW approaches. Similarly, both perspectives (micro and macro) could clarify the unifying or divisive characteristics of various marketing approaches.

Furthermore, it is reasonable to assume that there is room for a fruitful combination of BMBW approaches to solve wicked problems, where a combination of not only several stakeholders but also several approaches is needed. It may be of interest to look at wicked problems (for example, climate collapse). However, we do not exclude that there might be specific circumstances under which one approach is more suitable than the others.

Future research should also explain the axiology of marketing for good and better specify what “good” means and which perspective defines it. Bringing the discussion on ethics and virtue will give a new turn in marketing research, strengthening the sustainability of marketing practices.

### Avenue 2: integrating marketing definition

As widely accepted definition of marketing^1^ encompasses the importance of contributing to the betterment of society, it is reasonable to question at what point BMBW approaches end, and “simple” marketing starts. As the question is complex, future research should investigate the intensity of endorsing BW marketing across various marketing research domains (e.g., communication, strategy, product development, distribution). Accordingly, we believe it is worthwhile to investigate research questions such as the following:

- When and under what conditions can we call Better Marketing for a Better World just marketing?
- What institutional, cultural, and social barriers prevent the acceptance of Better Marketing for a Better World as a synonym for marketing? If any, how can we overcome them?

As the definition of marketing relies on the definition of value, as for “customers, clients, partners, and society at large,” future research may want to delineate what value means for various actors and how marketing research domains (e.g., communication, strategy, product development, distribution) respond to this notion of value. It may be so that some aspects of marketing research have already endorsed the BMBW philosophy, whereas others are late to accommodate it.

Naturally, we envision various barriers that will impede such an equation and we expect that these barriers may vary across research domains and contexts. Moreover, we expect the involvement of various disciplines (e.g., psychology or political science) that will bring novel insights into the intertwined process of barrier overcoming.

### Avenue 3: extending the practical implementation of better marketing for a better world through marketing capabilities

To contribute to a better world, organizations need to develop certain marketing capabilities or collective abilities that address social problems of today’s world from a practical point of view. Once developed, these capabilities will spur the development of products that provide long-term social well-being and ensure a sustainable competitive advantage. As such, we believe it is worthwhile to investigate research questions such as the following:

- What kind of marketing capabilities (existing and new ones) may account for better world marketing?
- How can organizations develop a Better Marketing for a Better World capability?
- What individual ability and skills underpin the development of the Better Marketing for Better World capabilities?

Organizations should apply better world marketing practices by developing appropriate marketing capabilities to reach a better world. Marketing capabilities epitomize how firms learn and exploit market knowledge and respond to market or environmental changes quickly and efficiently (Xu et al., 2018). We encourage future research to analyze what marketing capabilities can be useful to increase the sustainability of BMBW principles.

To provide better clarification on how to develop these capabilities, scholars may want to draw attention to three main building blocks or “microfoundations” that literature generally adopts to conceptualize organizational capabilities: individuals, processes, and structures (Felin et al., 2012). Particular attention should be given to the intersection of different building blocks to show how different organizational practices intertwine and how this intersection creates different collective abilities useful for using marketing practices for good.

In developing organizational capabilities, the individual level is the most important building block (Felin et al., 2012), as different human capital (skills, knowledge, experience, cognitive abilities) undoubtedly and unconditionally influence organizational modus operandi (Felin et al., 2012). We envision the development of new leaders’ and marketing managers’ skills that will eventually spur the development of collective abilities useful for advancing our society. For instance, the theory of individual mindfulness emphasizes that the ability to pay attention to one’s feelings and thoughts may translate into ethical awareness and a sense of social responsibility toward other people and the world (Nilsson & Kazemi, 2016).

## Conclusion

Today’s world is striving for a better application of marketing approaches. The purpose of this article is to critically review the most widely accepted approaches to BMBW and seek new research perspectives. Drawing on the main weaknesses or criticalities presenting in existing studies, this article contributes to identifying the shortcomings as the basis for future research on this topic. Notably, it provides associated future research agenda questions aimed at stimulating the reconceptualization of the marketing research domains and other related research disciplines. We anticipate that this refinement will strengthen the research focus on incorporating BMBW techniques into all research domains of marketing.

In addition to the theoretical implications, this article also provides implications for practice. Understanding BMBW and findings ways to enhance marketing for good would help organizations building a more sustainable business, which is crucial for their success in current times. This review may help organizations and policymakers develop strategies that generate real value for customers, thus contributing to the achievement of a more prosperous, fair, and sustainable society.

We acknowledge that our literature review does not fully and comprehensively explain our research topic as we based our analysis on selected approaches that are mostly associated with BMBW. Therefore, we cannot generalize the findings of our study to the whole subject field but our discussion may serve as an impetus for future researchers interested in bringing greater clarity to the topic from a theoretical perspective and strengthening its practical implementations.

## Footnotes

1 As defined by the American Marketing Association in 2017 “Marketing is the activity, set of institutions, and processes for creating, communicating, delivering, and exchanging offerings that have value for customers, clients, partners, and society at large”.
